# Effect of extended days on feed on carcass gain, efficiency, and quality of individually fed beef steers

**DOI:** 10.1093/tas/txae081

**Published:** 2024-05-08

**Authors:** Jessica L Sperber, Robby G Bondurant, Galen E Erickson, Kelly Bruns, Rick N Funston, Jim C MacDonald

**Affiliations:** Department of Animal Science, University of Nebraska, Lincoln, NE 68583-0908, USA; Department of Animal Science, University of Nebraska, Lincoln, NE 68583-0908, USA; Department of Animal Science, University of Nebraska, Lincoln, NE 68583-0908, USA; West Central Research and Extension Center, University of Nebraska, North Platte, NE 69101, USA; West Central Research and Extension Center, University of Nebraska, North Platte, NE 69101, USA; West Central Research and Extension Center, University of Nebraska, North Platte, NE 69101, USA

**Keywords:** backfat thickness, days on feed, hot carcass weight

## Abstract

Crossbred steers (*n* = 114, initial BW = 334 kg; SEM = 5 kg) were serially harvested to evaluate the change in carcass composition by feeding cattle 21 or 42 d longer than the 2014 industry average subjective measure of finish, 1.27 cm of 12th rib fat thickness. Carcass ultrasound measurements were collected on 76 steers at 1, 78, and 134 days on feed (DOF) to project appropriate harvest date. Steers were sorted into three harvest groups, and serially harvested at 142, 163, or 185 DOF, with the first harvest date selected based on an estimated 12th rib fat thickness of 1.27 cm via ultrasound measurement. Steers were fed using an individual animal feeding system, to determine individual performance metrics. Steer DMI did not differ (*P *≥ 0.31) between harvest groups, while carcass-adjusted ADG and G:F decreased linearly (*P* ≤ 0.04) as DOF increased. Carcass weight increased linearly (*P* < 0.01) as DOF increased from 142 to 185 DOF, with steers gaining an additional 36 kg of HCW when fed an additional 42 DOF. Carcass LM area quadratically increased (*P* = 0.04) to 163 DOF and remained constant to 185 DOF. Marbling score was not different (*P* = 0.14) between harvest groups; however, the opportunity to grade USDA Premium Choice was improved for steers fed to 185 DOF. Calculated YG and 12th rib fat thickness increased linearly (*P* < 0.01) as DOF increased, with distributions across YG 1 through 5 differing between harvest groups (*P* < 0.01), and 185-d carcasses having the greatest frequency of YG 4 carcasses. As cattle are fed for additional DOF, live ADG and G:F decline, while HCW and LM area increase.

## Teaser

Extending days on feed reduces average daily gain and feed efficiency in beef feedlot steers but increases hot carcass weight and opportunity to achieve higher quality grades for beef.

## Introduction

As the U.S. fed beef cattle prices increased in 2014 and reached historically high prices in 2015, feedlot operators increased average days on feed (DOF) in an attempt to capture additional revenue from heavier hot carcass weight (HCW). Optimal market endpoint for beef cattle can be described as the inflection point at which cost of additional gain equals the price received for the additional gain and has been extensively researched (Pyatt et al., 2005b; Streeter et al., 2012; Tatum et al., 2012; Wilken et al., 2015). As the industry increasingly markets on a carcass-basis rather than live basis, more consideration must be given to factors that affect optimal market endpoint. Previously, HCW has been determined to be the leading factor influencing profitability of feedlot cattle (Pyatt et al., 2005a, 2005b; Streeter et al., 2012; Tatum et al., 2012; Wilken et al., 2015). Hot carcass weight is largely dependent on length of the feeding period or number of DOF. As DOF increase, carcass transfer from live ADG can reach up to 88.6% for steers (Streeter et al., 2012). It is also developed in literature that as DOF increase and fat depots accumulate, there is an increased opportunity to capture a premium for higher USDA quality grade (QG), assuming an increase in marbling score (Pyatt et al., 2005b; Streeter et al., 2012; Tatum et al., 2012). Because the energy deposited as fat on the carcass remains with the animal through harvest (minus drop weight of hide, head, hooves, and offal), this weight in fat is transferred to HCW and adds value to the animal (Streeter et al., 2012). However, when selling cattle on a grid-based marketing system, there is a risk in taking cattle to heavier endpoints, as longer DOF is associated with heavier HCW and greater 12th rib fat thickness, increasing the risk of receiving discounts for overweight carcasses and USDA Yield Grade (YG) 4s and 5s (Pyatt et al., 2005b; Streeter et al., 2012).

The objective of this experiment was to evaluate the effects of extended DOF on live performance and carcass characteristics of individually fed finishing steers. Compositional changes in lean protein and fat deposition over the feeding period were evaluated using real-time carcass ultrasound techniques by measuring LM area, 12th rib fat thickness, and intramuscular fat percent (IMF).

## Materials and Methods

All animal care and management procedures were approved by the University of Nebraska-Lincoln Institutional Animal Care and Use Committee (IACUC # 823).

Yearling crossbred steers (*n* = 114, initial BW = 334 kg; SEM = 4.8 kg) were individually fed using a GrowSafe feeding system (GrowSafe Systems Ltd., Airdrie, AB, Canada) at the West Central Research and Extension Center in North Platte, Nebraska, from May to November 2014. The experiment utilized a randomized complete block design, with steers stratified by initial BW and randomly assigned within pen to one of three serial harvest date treatment groups. Treatments included steers harvested at a live fat endpoint of 1.27 cm (accurate for 2014 industry 12th rib fat thickness average), steers harvested 21 or 42 d longer than the number of DOF projected to achieve a fat endpoint of 1.27 cm. Therefore, treatments included steers harvested at 142, 163, and 185 DOF.

Before experiment initiation, steers were limit-fed 55% Sweet Bran (Cargill Corn Milling; Blair, NE) and 45% prairie hay (DM basis) for five consecutive days to minimize variation in gut fill as described by Watson et al. (2013). Following limit-feeding, steers were adapted to a common finishing diet for 24 d and were implanted with 80 mg trenbolone acetate and 16 mg estradiol (Revalor-IS; Merck Animal Health, Madison, NJ) on the second day of the adaptation period. Following the adaptation period, steers were moved into the GrowSafe feeding facility to measure individual DMI. Upon entry into the GrowSafe feeding facility, steers were weighed for two consecutive days to determine average initial BW and a 4% shrink was applied to equalize gut fill. Steers were then stratified by BW and assigned to treatment. The initial BW and performance metrics were calculated utilizing the 4% shrunk BW when steers were moved into the GrowSafe feeding facility, and therefore, the 24-d adaptation period to the finishing diet was not included in DOF calculation, and day 1 of the experiment began upon arrival into the GrowSafe facility. Steers were fed twice daily a common finishing diet to maintain ad libitum intake. The finishing diet contained 48% dry-rolled corn, 40% Sweet Bran, 7% prairie hay, and 5% supplement (DM basis; Table 1) and was loaded, mixed, and delivered via feed truck (Roto-mix model 414-14B; Roto-mix, Dodge City, KS). At 80 DOF, steers were administered an additional implant of 200 mg trenbolone acetate and 20 mg estradiol (Revalor-200, Merck Animal Health), which equated to 102 d after initial implant.

**Table 1.** Ingredient and nutrient composition of finishing diet fed to steers across days 142, 163, and 185 DOF

Ingredient	% of Diet DM
Dry-rolled corn	48
Sweet Bran^1^	40
Prairie hay	7
Supplement^2^	5
Nutrient analysis,% DM	
DM	71.94
Crude protein	13.8
Ca	1.09
P	0.54
NEm, Mcal/kg of DM	1.72
NEg, Mcal/kg of DM	1.04

^1^Sweet Bran (Cargill Corn Milling)

^2^Supplement formulated to provide a minimum of 13.5% CP, Ca:P of 2:1, 450 mg monensin (Rumensin 90, Elanco Animal Health, Indianapolis, IN), 90 mg tylosin (Tylan, Elanco Animal Health) daily. The vitamin and trace mineral premix was formulated to provide 1,500 IU of vitamin A, 3,000 IU of vitamin D, 3.7 IU of vitamin E per g daily, and contained 10% Mg, 6% Zn, 4.5% Fe, 2% Mn, 0.5% Cu, 0.3% I, and 0.05% Co (diet DM).

Real-time carcass ultrasound measurements including LM area, 12th rib fat thickness, and IMF were collected on 76 steers at 1, 78, and 134 DOF by a Centralized Ultrasound Processing (CUP Lab; Ames, Iowa) certified field technician. Images were captured using an Aloka 500-V unit (Corormetrics Medical Systems, Wallingford, CT) equipped with a 3.5-MHz, 17.2 cm linear array transducer. All images were captured on the right side of the steer. To capture 12th rib fat thickness and LM area, the steer was palpated to locate the 13th rib and the transducer was placed laterally between the 12th and 13th ribs, using a standoff guide to capture the image. Images for IMF prediction were collected by placing the transducer three-fourths the distance from the medial end of the LM area to the lateral end and horizontally over the 12th and 13th ribs. Ultrasound image interpretation was conducted by a certified technician at the CUP Lab. After interpretation, ultrasound IMF was converted to USDA marbling score using data presented by Wilson et al. (1998) to allow for comparisons with carcass data post-harvest. Total DOF for each serial harvest group were calculated as total days that feed was delivered to the bunk while cattle were fed from the GrowSafe bunks. Carcass camera data were collected by the commercial packing facility, Tyson Fresh Meats (Lexington, Nebraska).

It is well described in the literature that dressing percent increases with increasing DOF, so a common dressing percent was not applied to serial harvest groups for carcass-adjusted live performance. Instead, the actual dressing percent calculated for each respective group was utilized to calculate carcass-adjusted performance metrics. Due to limitations of the handling facility at the research station, individual steer live final BW prior to harvest was not collected, and therefore, the average dressing percent for each harvest group was calculated using the total HCW sold divided by the gross live weight (no shrink) of the 38 steers harvested for each serial harvest date. The un-shrunk gross live weight was collected upon arrival at the commercial abattoir. Incremental carcass average daily gain and G:F were calculated in an attempt to quantify performance over extended DOF on a carcass-basis. Carcass-based gain and G:F were calculated using the following equations:


$$\begin{array}{l} Carcass\;ADG\;for\;163\;DOF:\;\frac{163\;DOF\;average\;HCW-142\;DOF\;average\;HCW}{21\;DOF} \end{array}$$

$$\begin{array}{l} Carcass\;ADG\;for\;185\;DOF:\;\frac{185\;DOF\;average\;HCW-163\;DOF\;average\;HCW}{21\;DOF} \end{array}$$

$$\begin{array}{l} Carcass\;G:F\;for\;163\;DOF:\;\frac{163\;DOF\;carcass\;ADG}{average\;DM\;intake\;from\;142\;to\;163} \end{array}$$

$$\begin{array}{l} Carcass\;G:F\;for\;185\;DOF:\;\frac{185\;DOF\;carcass\;ADG}{average\;DM\;intake\;from\;163\;to\;185} \end{array}$$

### Statistical Analyses

All live performance and carcass data were analyzed using the GLIMMIX procedure of SAS (SAS Institute, Inc., Cary, NC) with steer as the experimental unit and pen included as a random effect. Covariate regression was utilized on ultrasound measured steers to develop 12th rib fat thickness, marbling score, and LM area data points. The model evaluated the effect of DOF for each variable. Orthogonal contrasts were used to test linear and quadratic effects of increasing DOF for steers. Categorical data, including YG and QG, were analyzed using the GLIMMIX procedure of SAS applying the logit link function (Stroup, 2013) with pen included as a random effect, steer as experimental unit, and the effect of DOF was determined. Significance was considered at α ≤ 0.05 and a tendency was considered at 0.05 < α ≤ 0.10.

## Results and Discussion

### Live Cattle Performance

There was no difference in initial BW for steers between treatment groups (*P* = 0.98) with steers averaging 334 kg at experiment initiation. Steer DMI was not impacted (*P *= 0.59) by increasing DOF from 142 to 185 DOF, an additional 42 DOF (Table 2). Streeter et al. (2012) reported similar findings for large pen studies where increasing DOF did not impact DMI, in addition, Galyean et al., (2023) found that extending DOF did not impact DMI for beef heifers in a 6-study summary. These findings are contrary to Hicks et al. (1987), Pyatt et al. (2005b), Wilken et al. (2015) who reported linear increases in DMI as DOF increased for finishing cattle. Results from a 7-study summary conducted on beef steers, reported a slope of 0.0025, suggesting that for each additional DOF, steer DMI would increase by 0.0025 kg (Galyean et al., 2023). Kirkpatrick et al. (2023) reported a quadratic reduction in DMI as steers were fed to 378 DOF. Dry matter intake is an important performance measure, as it affects incremental cost of gain (COG), accounting up to 3% of variation in profit among steers due to substantial influence on COG (Pyatt et al., 2005b). Variations on the effect of extended DOF on DMI could be due to time of year cattle are nearing market endpoint, the affect that environment may have on intake, cattle weight, breed, health, diet composition, and various other factors.

**Table 2.** Feedlot and carcass performance of individually fed steers for 142, 163, and 185 days on feed

	Days on feed^1^				Contrasts^2^	
Characteristics	142	163	185	SEM	Linear	Quadratic
Initial BW, kg	333	334	334	4.8	0.98	0.96
DMI, kg/d	10.8	10.7	10.9	0.2	0.59	0.31
Carcass-Adj. Final BW^3^, kg	593	616	651	9.0	<0.01	0.56
Carcass-Adj. ADG, kg	1.70	1.56	1.54	0.05	0.06	0.37
Carcass-Adj. G:F, kg/kg	0.164	0.153	0.147	0.002	<0.01	0.30
HCW, kg	374	388	410	5.5	<0.01	0.56
LM area, cm^2^	89.0	93.5	92.3	1.3	0.06	0.04
Marbling score^3^	475	476	506	15	0.14	0.42
12^th^ rib fat, cm	1.24	1.47	1.75	0.10	<0.01	0.79
Calculated yield grade^4^	2.89	3.05	3.56	0.16	<0.01	0.20

^1^Steers fed to 142, 163, and 185 days on feed (*n* = 38 per treatment). Day 142 steers harvested at live animal industry average 12th rib fat depth of 1.27 cm.

^2^ *P*-values for preplanned linear and quadratic contrasts.

^3^Carcass-adjusted final body weight: HCW divided by actual dressing percent from each serial harvest time point (64.83%, 65.91%. and 66.14%, respectively).

^4^Marbling score: 400 = Small^00^, minimum required for USDA Low Choice; 500 = Modest^00^, minimum required for USDA Premium Choice.

^5^Calculated yield grade = 2.5 + (0.98 × 12th rib fat thickness, cm)—(0.05 × LM area, cm^2^) + (0.2 × KPH,%) + (0.0084 × HCW, kg; USDA , 1997).

Steer final BW, ADG, and feed efficiency were all reported on a carcass-adjusted basis, using the average dressing percent at harvest for each treatment group (Table 2). Average dressing percent at harvest was 63.5%, 64.6%, and 64.8% for 142, 163, and 185 d fed cattle, respectively. These values are consistent with equations derived by Bruns et al. (2004) and Wilken et al. (2015) where dressing percent increased as a function of increased HCW. Carcass-adjusted final BW increased linearly (*P <* 0.01) from 593 kg (142 DOF) to 651 kg (185 DOF) as steers were fed for an additional 42 DOF (Table 2). Steers fed for 21 and 42 d longer (harvested at 163 and 185 DOF) gained 23 and 58 kg more than steers harvested at 142 DOF, respectively. Carcass-adjusted ADG tended to decrease linearly (*P *= 0.06) from 1.70 to 1.54 kg/d when steers were fed for an additional 42 DOF. When increasing DOF from 142 to 163, carcass-adjusted ADG reduced 8.2% or 0.14 kg/d, with an additional reduction in carcass-adjusted ADG of 1.3% or 0.02 kg/d when increasing DOF from 163 to 185 d. The carcass-adjusted ADG reported in the current experiment was greater compared to large pen data described by Streeter et al. (2012) where steers gained 1.11 kg/d in the last 42 DOF. Results from a 7-study summary conducted on beef steers to determine the effect of extended DOF (up to 62 additional days) on cattle performance, reported a slope of −0.0024 for ADG, suggesting that for each additional DOF, steer rate of gain would reduce by 0.0024 kg (Galyean et al., 2023). Carcass-adjusted G:F decreased linearly (*P < *0.01) from 0.164 to 0.147 kg/kg as steers were fed to 142 or 185 DOF, respectively. Feed efficiency reduced 6.7% when increasing DOF from 142 to 163 d, and reduced an additional 3.9% when DOF increased from 163 to 185 d. The concept that live ADG and G:F decrease as DOF increase has been thoroughly depicted in literary works. Pyatt et al. (2005b), Streeter et al. (2012), and Wilken et al. (2015) described linear reductions in live ADG and G:F finishing cattle were fed for longer DOF. Similarly, Martinez et al. (2023) fed steers in a commercial feedlot setting to increasing DOF, reporting a reduction in ADG of 0.08 kg/d or 4.4% and G:F of 0.011 kg/kg or 6.2% as DOF increased from 166 to 194, reporting no additional reduction in ADG and G:F as steers were fed to 208 DOF when compared to 194 d fed cattle. Kirkpatrick et al. (2023) completed a serial slaughter study on steers fed extended DOF, up to 378 d, reporting a quadratic reduction in ADG and G:F. These data suggest that the reduction in ADG and G:F as DOF increases, may follow a linear decline, until a certain point, in which the reduction in live performance flat-lines.

### Ultrasound Performance

**Figure 1.**
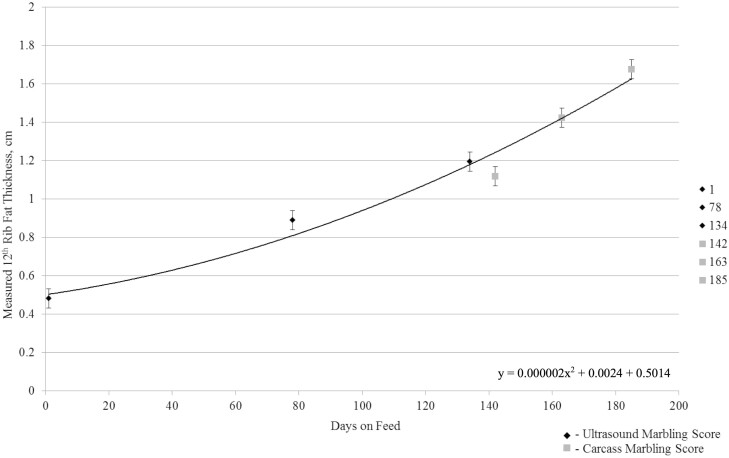
Measured 12th rib fat thickness (cm) throughout days on feed. On days 1, 78, and 134, backfat thickness was measured on two pens of cattle (*n* = 76) using real-time carcass ultrasound, with the average of the two pens reported. Steer 12th rib fat thickness was measured again on days 142, 163, and 185 at harvest for each serial slaughter group (*n* = 38 per harvest date) and averaged. Steer 12th rib fat thickness increased quadratically (*P *< 0.01) from 0.48 cm at day 1 to 1.75 cm at 185 DOF.

**Figure 2.**
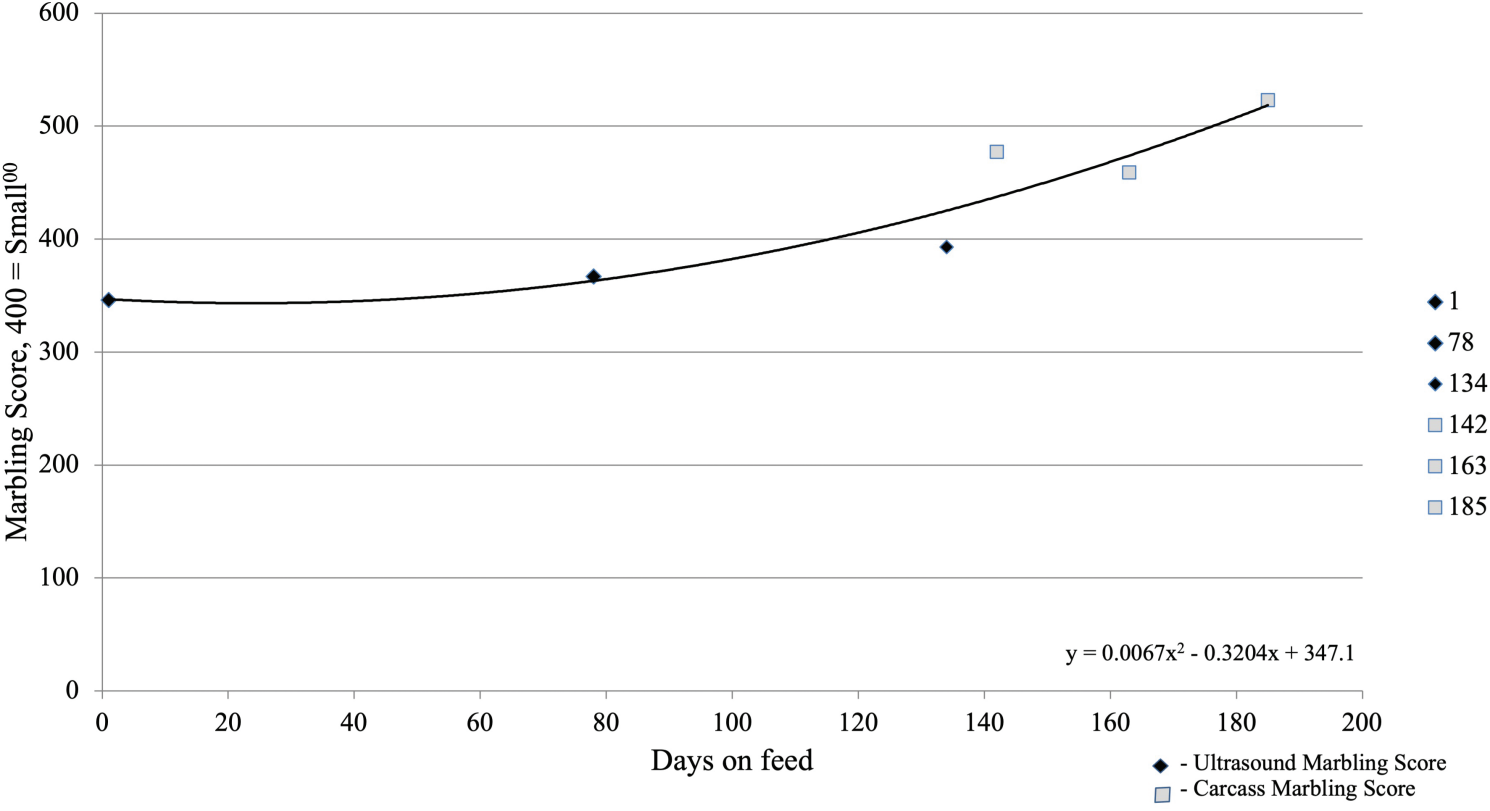
Marbling score of steers across increasing days on feed. Intramuscular fat was measured on days 1, 78, and 134 using real-time carcass ultrasound on two pens of cattle (*n* = 76) and averaged. Ultrasound measurement was evaluated as percent intramuscular fat and converted to marbling score (Wilson et al., 1998). Marbling score for days 142, 163, and 185 was evaluated at harvest for each serial slaughter group (*n* = 38 per harvest date) and averaged. Marbling score quadratically increased (*P *< 0.01) from day 1 at 346 to 526 at 185 DOF.

**Figure 3.**
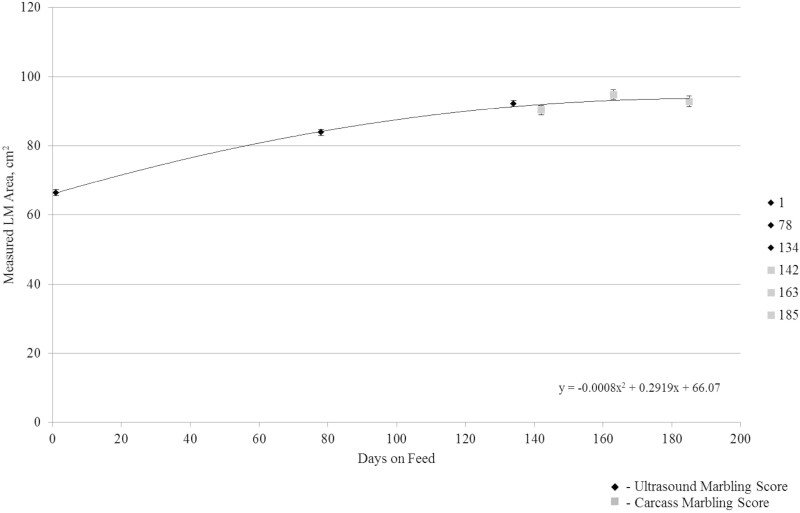
Measured LM area (cm^2^) of steers across increasing days on feed. Measured LM area on days 1, 78, and 134 using real-time carcass ultrasound on two pens of cattle (*n* = 76) and result was averaged. Longissimus muscle area was measured on days 142, 163, and 185 at time of harvest for each serial slaughter group (*n* = 38 steers per harvest date) and averaged. Steer LM area increased quadratically (*P *< 0.01) from 66.5 cm^2^ at day 1 to 92.3 cm^2^ at 185 DOF.

On day 1 when initial ultrasound was conducted, 12th rib fat thickness, IMF converted to marbling score, and LM area were not different (*P ≥* 0.42) among harvest groups. Steer 12th rib fat thickness increased quadratically (*P <* 0.01) from 0.48 cm on days 1 to 1.65 cm on day 185 (Figure 1). Steers ultrasounded at 134 DOF had 1.19 cm rib fat, while those harvested at 142 DOF had a carcass 12th rib fat thickness of 1.12 cm. This small discrepancy, reduction of 0.07 cm, in fat thickness between the ultrasound measurement and at harvest may be due to subsequential external fat loss during hide pulling and carcass trim at the commercial abattoir or from the inherent differences in ultrasound and camera measurements. May et al. (1992) and Bruns et al. (2004) similarly described quadratic increases in 12th rib fat thickness as cattle were fed for longer DOF. Marbling score increased quadratically (*P <* 0.01) from 346 at initial ultrasound on days 1 to 523 at day 185 (Figure 2), consistent with findings described by May et al. (1992) where marbling score increased as a function of increasing DOF. Ultrasound measured LM area increased quadratically (*P <* 0.01) from 66.5 cm^2^ at initial ultrasound on day 1 to 92.9 cm^2^ as steers were fed to 185 DOF (Figure 3). Others have reported on the quadratic effects of DOF on LM area (Hicks et al., 1987; May et al., 1992; Bruns et al., 2004; Streeter et al., 2012) in which LM area of cattle increases linearly, until the animal has reached physiological maturity, at which point the rate of LM area begins to increase at a decreasing rate.

### Carcass Characteristics

As steers were fed from 142 to 185 DOF, HCW increased linearly (*P <* 0.01; Table 2), adding an additional 14 kg of HCW for the first 21 d increment (from 142 to 163 DOF), and an additional 22 kg for the second 21 d increment (from 163 to 185 DOF), totaling 36 kg of additional HCW when steers were fed for 42 additional days. These results follow a similar trend with Streeter et al. (2012) where steers gained 17 and 20 kg of additional HCW for each 21-d incremental increase in DOF. Carcass-based ADG and G:F were calculated using the equations reported in the materials and methods. Incremental carcass-based ADG increased as steers were fed to longer DOF, where steers fed an additional 21 d from days 142 to 163 had a carcass ADG of 0.67 kg/d, and steers fed from 163 to 185 DOF had a carcass ADG of 1.05 kg/d. Carcass-based G:F was 0.062 between days 142 and 163, and 0.097 between days 163 and 185, exemplifying an increasing rate of carcass gain as DOF increases. The increased carcass gain and G:F as DOF increased can be alluded to an increase in carcass transfer, illustrated by the increase in DP as DOF increased, which is calculated as the kilogram of live weight gain that results on the carcass at time of slaughter. Streeter et al. (2012) reported a slightly greater rate of carcass rate of gain at 0.95 kg/d for steers while Wilken et al. (2015), using regression of pooled data, described carcass gain as increasing at a decreasing quadratic rate as DOF increased and May et al. (1992) reported carcass gain increasing linearly as DOF was increased.

Steer LM area quadratically increased (*P =* 0.04; Table 2) from 89.0 to 93.5 cm^2^ (142 and 163 DOF, respectively) and decreased slightly to 92.3 cm^2^ at 185 DOF. Data from previous studies reported linear increases in LM area as HCW increased, however, LM area as a percentage of HCW gain decreased with increasing DOF (Hicks et al., 1987; May et al., 1992; Bruns et al., 2004; Streeter et al., 2012). Galyean et al. (2023) reported that LM area tended to increase with extended DOF for steers in a 7-study summary and increased with extended DOF for heifers in a 6-study summary.

**Figure 4.**
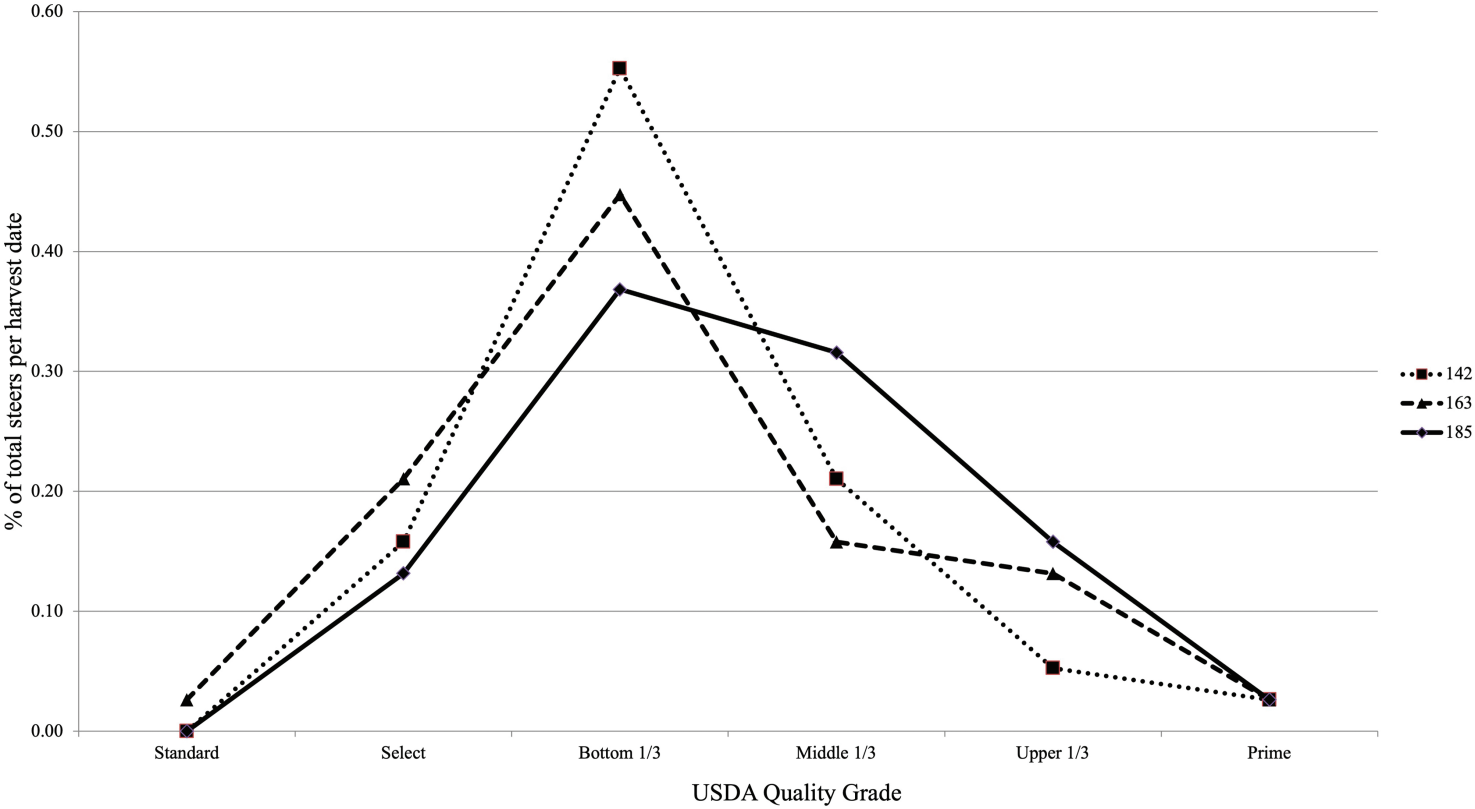
Percent of total steers harvested (38 steers per harvest date) on day 142 (dotted line), 163 (dashed line), and 185 (solid line) having USDA quality grade of Standard, Select, bottom 1/3 Choice, middle 1/3 Choice, upper 1/3 Choice, and Prime. Although there was an increase in the percentage of steers grading upper 2/3 Choice for 185 d, there was no difference in USDA quality grade (*P =* 0.18) as steers were fed increasing days on feed.

Carcass marbling score (*P =* 0.14) and USDA quality grade (*P =* 0.18) did not differ as additional DOF were added; however, marbling score did numerically increase when steers were fed for an additional 21 d, from 163 to 185 DOF (Table 2). Carcasses fed to 185 DOF averaged a marbling score of 506, qualifying 185 d carcasses to grade USDA Premium Choice, with 163 d carcasses averaging a marbling score of 476, qualifying 163 d carcasses to grade USDA commodity Choice (Figure 4). Biologically, marbling score increases as a function of increased fat deposition as cattle are fed for extended DOF. However, like the current experiment, Brandt et al. (1992) and Haugen et al. (2004) reported that DOF had no effect on marbling score. Others have described a linear increase in marbling score (Bruns et al., 2004; Pyatt et al., 2005b) or a quadratic increase in marbling score (May et al., 1992) as DOF increase. Galyean et al. (2023) reported that extending DOF in finishing steers and heifers raised marbling score, and Martinez et al. (2023) reported an increase in marbling score for steers on feed for an additional 42 d. Numerical results from Martinez et al. (2023) were consistent with the marbling response reported in the current experiment, where steers gained an additional 25 points of marbling over 28 DOF, increasing the opportunity for 208 d fed cattle to grade USDA Premium Choice.

Carcass 12th rib fat thickness increased linearly (*P <* 0.01) from 1.24 to 1.75 cm as DOF increased from 142 to 185 d, with an intermediate 12th rib fat thickness of 1.47 cm for 163 d steers (Table 2). Similar to the current experiment, linear increases in 12th rib fat thickness as a function of increased DOF has been reported by May et al. (1992) and Van Koevering et al. (1995). However, differing from the results of the current experiment, Hicks et al. (1987) and Bruns et al. (2004) described quadratic increases in 12th rib fat thickness as DOF increased. Bruns et al. (2004) attributed the difference between the linear and quadratic response to DOF to a point of inflection for 12th rib fat thickness rate of gain in which a plateau in additional back fat deposition was reached when a quadratic response was noted. Martinez et al. (2023) reported a linear increase in 12th rib fat thickness over 42 additional DOF, with fat thickness measuring 1.78 cm for steers fed to 194 DOF. Interestingly, 12th rib fat thickness reported by Martinez et al. (2023) for cattle fed to 194 DOF (1.78 cm) was similar to the current experiment, where 12th rib fat thickness for cattle fed to 185 DOF measured 1.75 cm. Although the current experiment and Martinez et al. (2023) were conducted nearly a decade apart, the impact of extended DOF on 12th rib fat thickness was parallel. Although feeder cattle have trended toward longer DOF, heavier HCW, and improved QG over the last 10 years, the similarity in 12th rib fat thickness measure between Martinez et al. (2023) and the current experiment are alluded to genetic improvements, where cattle are later maturing to yield a bigger carcass, and the adoption of fat repartitioning agents such as beta-agonists.

**Figure 5.**
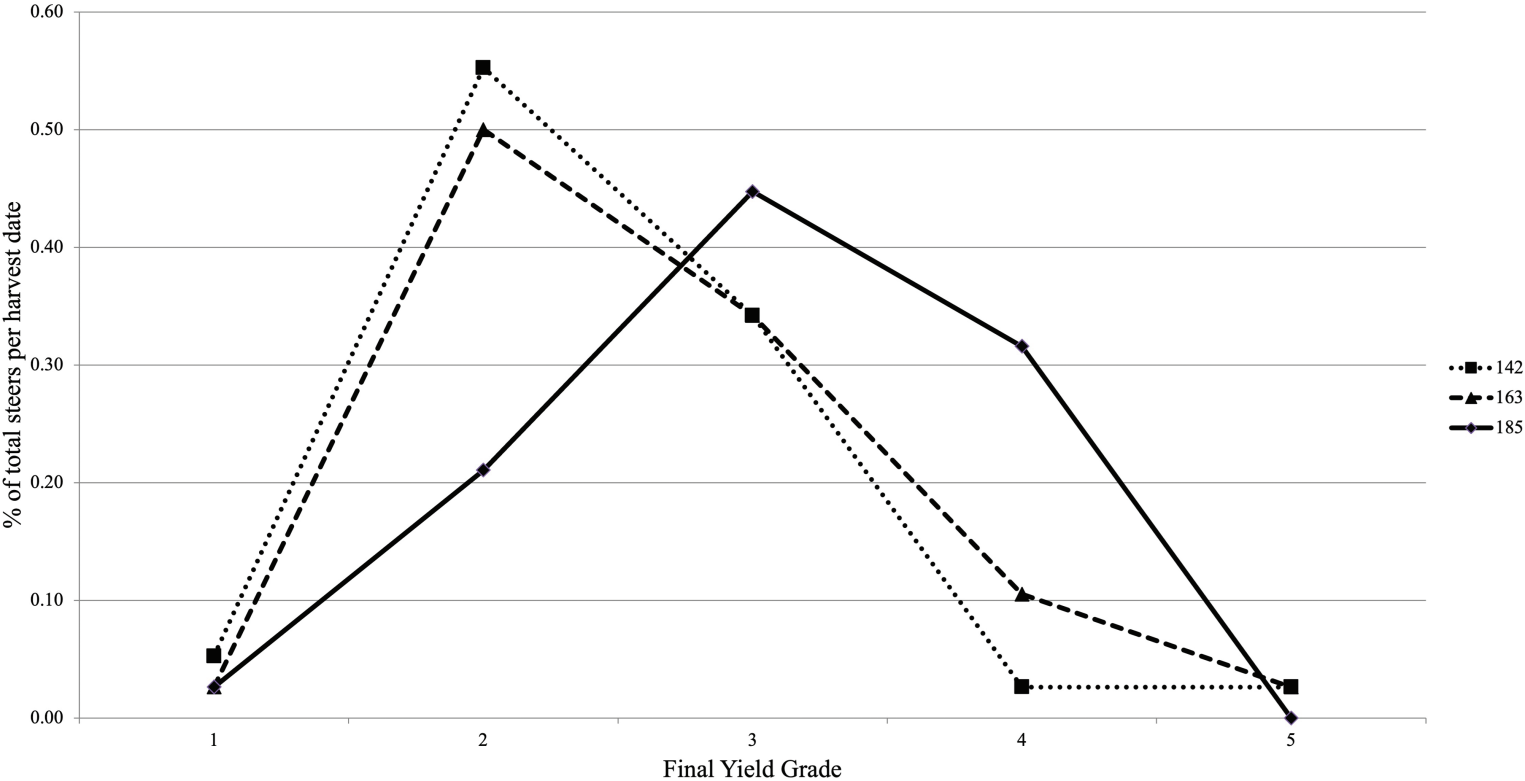
Frequency of YG 1 through YG 5 for steers harvested (38 steers per harvest date) on days 142 (dotted line), 163 (dashed line), and 185 (solid line). Final YG was increased (*P *< 0.01) with increasing days on feed from days 142 to 185, where steers fed 185 DOF had the greatest percent YG 3 and 4.

As steers deposited greater fat with extended DOF, calculated YG linearly increased (*P <* 0.01; Table 2) from YG 2 to YG 3 when steers were fed to 185 DOF. The distribution of carcasses that graded YG 1 through YG 5 were impacted by DOF (Figure 5; *P *< 0.01). Steers harvested at 185 DOF had an additional 10.5% YG 3 carcasses, compared to steers harvested at 142 and 163 DOF, which did not differ. The frequency of YG 4 carcasses increased from 2.6% to 10.5% and 31.6% as DOF increased from 142 to 163 and 185, respectively (Table 3). Interestingly, there were no YG 5 carcasses for the 185-d harvest group; however, 2.6% of the steers for 142 and 163 d harvest groups measured YG 5. It has been well documented in the literature that YG increases linearly as DOF increases, as a function of increased HCW and greater 12th rib fat thickness (Haugen et al., 2004; Pyatt et al., 2005b; Streeter et al. 2012). When marketing on a grid basis, frequency of YG 4 and 5 carcasses become a factor when determining optimal market endpoint to minimize YG discounts while increasing revenue generated from HCW (Tatum et al., 2012). Many researchers have concluded QG improvements, attributed to extended DOF, have a positive return when marketing on a grid; however, the determining factor on feeding increased DOF is ultimately HCW and the associated discount for overweight carcasses (Fuez, 2002; Pyatt et al., 2005a; Streeter et al., 2012; Tatum et al., 2012). Optimal feeding endpoint occurs when additional carcass weight gain (HCW) from increased DOF overcomes the YG 4, YG 5, and heavyweight carcass discounts.

**Table 3.** Frequency of USDA carcass quality and yield grade outcomes of steers serially harvested across increasing days on feed

	Days on feed^1^						
	142		163		185		
Item	Mean	SEM	Mean	SEM	Mean	SEM	*P*-value
QG,%							0.18
Prime	2.63	0.85	2.63	1.10	2.63	0.55	—
Upper 1/3 Choice	5.26	1.30	13.16	1.23	15.79	2.32	—
Middle 1/3 Choice	21.05	3.78	15.79	3.63	31.58	5.50	—
Bottom 1/3 Choice	55.26	6.92	44.74	6.81	36.84	7.64	—
Select	15.79	5.48	21.05	5.64	13.16	3.74	—
Standard	0	1.04	2.63	1.10	0	0.55	—
YG,%							<0.01
1	5.26	3.03	2.63	2.18	2.63	0.73	—
2	55.26	9.70	50.00	9.97	21.05	7.60	—
3	34.21	3.41	34.21	4.51	44.74	8.52	—
4	2.63	0.56	10.53	0.81	31.58	2.46	—
5	2.63	3.03	2.63	2.18	0.00	0.73	—

^1^Steers fed to 142, 163, and 185 d on feed (*n* = 38 per treatment). Day 142 steers harvested at live animal industry average 12th rib fat depth of 1.27 cm.

Consideration should be given to the fact these steers were not fed a β-agonist in the current experiment. Researchers have reported that β-agonists increase LM area, DP, and HCW, while decreasing YG when compared to a negative control (Ricks et al., 1984; Avendaño-Reyes et al., 2006; Vasconcelos et al., 2008; Elam et al., 2009) by repartitioning energy to protein accretion as opposed to fat deposition. Feeding a β-agonist toward the end of the feeding period could aid in minimizing discounts for YG 4 and 5 carcasses while also adding HCW to capture additional revenue. Additionally, β-agonists have been reported to negatively affect QG for cattle fed similar DOF (Ricks et al., 1984; Avendaño-Reyes et al., 2006; Vasconcelos et al., 2008; Elam et al., 2009), which could affect possible premiums received for increased marbling.

## Implications

Although carcass-adjusted live ADG and G:F decreased with increasing days on feed, steer HCW increased by 14 and 36 kg with 21 or 42 additional DOF compared to the 2014 industry average of selling at 1.27 cm 12th rib fat thickness. The loss associated with reduced live performance in terms of ADG and G:F, may be overcome by the opportunity to increase final BW and HCW at time of marketing. As cattle were fed for longer DOF, an increase in the proportion of carcasses that graded YG 4s and 5s increased, with no subsequent improvement in marbling score and QG. If market conditions offer reduced feed cost and cost-effective feeder purchase price, steers can be fed for extended DOF and sold on a carcass-basis to capture profit potential for heavier HCW.
